# Dose-dependent genotype effects of BDNF Val66Met polymorphism on default mode network in early stage Alzheimer's disease

**DOI:** 10.18632/oncotarget.11027

**Published:** 2016-08-02

**Authors:** Pin-Hsuan Lin, Shih-Jen Tsai, Chi-Wei Huang, Liu Mu-En, Shih-Wei Hsu, Chen-Chang Lee, Nai-Ching Chen, Ya-Ting Chang, Min-Yu Lan, Chiung-Chih Chang

**Affiliations:** ^1^ Department of Health and Beauty, Shu-Zen College of Medicine and Management, Kaohsiung, Taiwan; ^2^ Psychiatric Department of Taipei Veterans General Hospital, Taipei, Taiwan; ^3^ Psychiatric Division, School of Medicine, National Yang-Ming University, Taipei, Taiwan; ^4^ Department of Radiology, Kaohsiung Chang Gung Memorial Hospital, Chang Gung University College of Medicine, Kaohsiung, Taiwan; ^5^ Department of Neurology, Cognition and Aging Center, Kaohsiung Chang Gung Memorial Hospital, Chang Gung University College of Medicine, Kaohsiung, Taiwan

**Keywords:** Alzheimer's disease, genetic dosage effect, anatomical structural covariance, default mode network, brain-derived neurotrophic factor, Gerotarget

## Abstract

In humans, brain-derived neurotrophic factor (BDNF) has been shown to play a pivotal role in neurocognition, and its gene contains a functional polymorphism (Val66Met) that may explain individual differences in brain volume and memory-related activity.

In this study, we enrolled 186 Alzheimer's disease (AD) patients who underwent 3D T1 magnetic resonance imaging, and explored the gray matter (GM) structural covariance networks (SCN). The patients were divided into three groups according to their genotype: Met/Met (*n* = 45), Val/Met (*n* = 86) and Val/Val (*n* = 55). Seed-based analysis was performed focusing on four SCN networks. Neurobehavioral scores served as the major outcome factor.

Only peak cluster volumes of default mode medial temporal lobe network showed significant genotype interactions, of which the interconnected peak clusters showed dose-dependent genotype effects. There were also significant correlations between the cognitive test scores and interconnected-cluster volumes, especially in the orbitofrontal cortex.

These findings support the hypothesis that BDNF rs6265 polymorphisms modulate entorhinal cortex-interconnected clusters and the valine allele was associated with stronger structural covariance patterns that determined the cognitive outcomes.

## INTRODUCTION

Neurotrophins are a group of molecules which play a key role in regulating neural survival, development and maintenance. The most abundant neurotrophic factor is brain-derived neurotrophic factor (BDNF) [1, 2] and has been reported to be of clinical significance in mediating the hippocampus network function [3, 4]. Activity-dependent BDNF secretion has been reported to be required for long-term potentiation and depression [5, 6] while impaired patterns of discrimination and learning deficits have been observed in BDNF knockout mice [7]. The salient pathological feature in Alzheimer's disease (AD) is hippocampal network degeneration, and BDNF has been shown to play a protective role in attenuating amyloid-related toxicity [5, 8].

In humans, the BDNF gene has been mapped to chromosome 11p14.1. A common single nucleotide polymorphisms (SNP) consisting of a missense change of the coding exon at position 66 (Val66Met, rs6265) has been shown to produce non-conservative amino acid changes (Val to Met). The Met allele carriers have been shown to have poorer episodic memory, and this may be reflected in lower activities of BDNF with impaired neuronal processing and trafficking [9]. A recent meta-analysis study [10] showed no significant associations between the BDNF Val66Met polymorphism and the risk of developing AD in dominant (Met *vs*. Val/Val), recessive (Met/Met *vs*. Val) and additive (Met/Met *vs*. Val/Val) carriers. Another meta-analysis reported that the Met66 allele conferred susceptibility to AD in women but not in men [11].

The influence of BDNF polymorphisms on human cognition has been linked with its impact on changes in brain structure. However, whether the Met allele leads to worse outcomes is controversial. Smaller brain volumes have been reported in Met carriers in the hippocampal and dorsolateral prefrontal areas [12], hippocampus [13], hippocampal, amygdala, thalamus, fusiform gyrus and frontal gyrus [14]. Other studies have reported no significant differences in hippocampal volume, whole brain volume or memory scores between Met carriers and Val homozygotes [15], and no significant genetic effects of BDNF on cognitive performance [16]. A protective role of the Met allele in regional gray matter (GM) volume and neurocognitive performance have been reported [17–19]. Given the role of BDNF as a crucial mediator in the maintenance of neuronal function, it is not known whether the Val66Met genotype is related to large-scale network modulation in patients with AD.

Recent research has suggested that highly related regions show covariance in morphometric characteristics, so called structural covariance. Structural covariance networks (SCNs) can be used to test the influence of the genotype with careful control of other factors. Three SCNs have been reported to be relevant to patients with AD: default mode network (DMN) [20–22], salience network [23] and executive control network [24, 25]. A recent report suggested that the DMN may be comprised of multiple, spatially dissociated but interactive components [26], of which two subsystems are particularly relevant: the “medial temporal lobe subsystem”, and the “dorsal medial prefrontal cortex subsystem” (or the midline core subsystem).

The potential mechanisms of genetic-based neurobiology are still under investigation, however a number of studies have highlighted how genetic variations [27–30], epigenetic [31, 32] or metabolic interactions [33] may affect organization of the brain or therapeutic programs [34]. In this study, we hypothesized that BDNF Val66Met functional polymorphisms may modulate the large-scale structural covariance pattern in patients with AD, and that network alterations may also determine the neurobehavioral characteristics.

## RESULTS

### Demographic data, cognitive data and NPI

The demographic characteristics and neuropsychiatric test results of the three genotype groups are listed in Table 1. The Val66Met genotype distribution was in Hardy-Weinberg equilibrium. There were no significant differences in apolipoprotein E4 allele distribution, MMSE scores, total or individual CASI scores, NPI total scores or levels of cerebrovascular risk biomarkers among the groups.

**Table 1:** Demographical characteristics and neuropsychiatric tests in the BDNF A homozygotes, G homozygotes and GA in Alzheimer's disease

Group	A homozygotes (*n* = 45)	GA (*n* = 86)	G homozygotes (*n* = 55)	*P* value
Age	73.6(6.6)	73.4 (8.8)	73.5(8.6)	0.99
Education (year)	6.6 (4.9)	7.3(5.1)	6.4 (5.1)	0.56
Apolipoprotein E4 carrier (positive case, %)	19, 42.2 %	33,38.4 %	25, 45.5 %	0.65
Sex (male/female)	18/27	46/40	18/37	0.44
Mini-Mental State Examination	19.5 (6.0)	20.2 (7.0)	17.9 (6.5)	0.12
CASI total scores	64.7 (21.6)	67.5(25.0)	60.7 (21.5)	0.24
Short Term Memory	4.5 (3.9)	5.6(3.9)	4.1(3.6)	0.05
Orientation	11.9 (5.3)	12.6 (5.7)	10.6 (5.3)	0.11
Long Term Memory	8.1(2.9)	8.2 (2.8)	8.11(2.7)	0.98
Language	7.7(2.4)	7.9(2.6)	7.72(2.3)	0.94
Drawing	7.67(3.1)	7.71(3.4)	7.20(3.1)	0.64
Executive function test scores	25.0 (8.9)	25.2(9.4)	23.2(8.4)	0.40
Neuropsychiatric inventory total scores	9.0 (13.0)	7.2 (11.4)	9.6(10.7)	0.45
Cerebrovascular Risk Biomarkers				
Homocysteine (umol/L)	13.35(6.29)	14.69(13.04)	12.63(4.05)	0.46
Hemoglobin-A1C (%)	6.21(1.67)	6.23(1.34)	9.61(1.21)	0.99
Creatinine (mg%)	1.05(0.34)	1.08(0.50)	0.96(0.37)	0.31
high-density lipoprotein (mg/dl)	61.38(17.34)	54.56(15.12)	55.46(13.37)	0.06
low-density lipoprotein (mg/dl)	105.35(40.14)	105.50(34.58)	113.35(41.49)	0.46
Total Cholesterol (mg/dl)	194.13(42.39)	182.05(37.25)	197.46(39.61)	0.06
Triglyceride (mg/dl)	115.90(68.46)	114.13(52.08)	125.40(69.09)	0.57
Hemoglobin (g/dl)	13.39(1.66)	13.43(1.80)	13.27(1.34)	0.86
Vitamin B12 (pg/dl)	686.03(363.42)	685.17(414.37)	689.45(383.59)	1.00
Folate (ng/dl)	15.90(17.53)	13.34(5.33)	14.03(5.76)	0.42

Data are presented as mean (standard deviation) or number (percentage; %)

Abbreviations: CASI, Cognitive Ability Screening Instrument; Attention, verbal fluency, abstract thinking, and mental manipulation sub-domain scores of the CASI were used to assess executive function. BDNF, brain APOE4 carriers were defined as the presence of one or two APOE4 alleles.

### Patterns of structural associations in the patients and genetic variants

**Figure 1:**
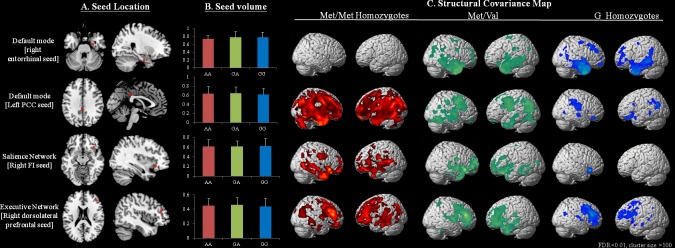
Statistical maps depicting brain areas in which the gray matter intensity covaried with four target seeds A. or seed volumes, and B. for separate structural covariance networks C. in all Alzheimer disease patients with the BDNF Met/Met (*n* = 45), Met/Val (*n* = 86) and Val/Val (*n* = 55) genotypes There was no significant difference in seed volume among the three genotypes (*p* > 0.05). Z-statistic maps (*p* < 0.05, corrected with a false discovery rate with extended cluster voxels > 100). The images are displayed on a standard brain render.

Among the three BDNF genotypes and four seed regions (Figure 1A), there were no significant differences in the GM volumes of each seed (Figure 1B). Networks showing structural associations with the seed regions for each genotype are shown in Figure 1C and Supplementary Table 1-12.

In the entorhinal seed-based DMN networks, the Met/Met homozygotes showed no clusters with a voxel size > 100 (Supplementary Table 1), while the Val/Met (voxel size = 53,366; Supplementary Table 2) and Val/Val homozygotes (voxel size = 49,132; Supplementary Table 3) showed clusters located in the para-hippocampus and sub-regions in the lateral temporal or frontal regions. In the DMN PCC seeds, the Met/Met homozygotes showed the greater numbers of voxels (voxel size = 167,815; Supplementary Table 4) compared with the Val/Met group (voxel size = 81,020; Supplementary Table 5) and Val/Val homozygotes (voxel size = 26,488; Supplementary Table 6).

For the frontoinsular seed, the Met/Met homozygotes (voxel size = 47,064; Supplementary Table 7) and Val/Met group (voxel size = 81,020; Supplementary Table 8) showed more extended voxels than the Val/Val homozygotes (voxel size = 1,142; Supplementary Table 9). For the dorsolateral prefrontal seed, the Met/Met homozygotes showed significantly greater structural covariance in the executive network (voxel size = 58,075; Supplementary Table 10) than the Val/Met group (voxel size = 35,720; Supplementary Table 11) and Val/Val homozygotes (voxel size = 19,225; Supplementary Table 12).

### Seed region volumes and relationships with the cognitive scores

We first explored whether each seed region volume was correlated with the selected cognitive test (Supplementary Figure 1). The DMN entorhinal (Supplementary Figure 1A) and PCC (Supplementary Figure 1B) seed volumes both showed significant correlations with the MMSE scores, CASI total scores, short-term memory, orientation and EFT scores, while the PCC seed also showed significant correlations with CASI language and drawing scores. For the salience network, only the CASI short-term memory score showed a significant correlation with the frontoinsular seed volume (Supplementary Figure 1C). For the executive control network, the seed region volume showed significant correlations with all test scores (Supplementary Figure 1D). The correlations between the seed volume and test scores suggested greater clinical significance of the PCC seed and dorsolateral prefrontal seed on the prediction of overall cognitive test scores.

### Peak clusters showing significant interactions between genotypes (Val/Val > Met/Met or Val/Met > Met/Met)

**Figure 2:**
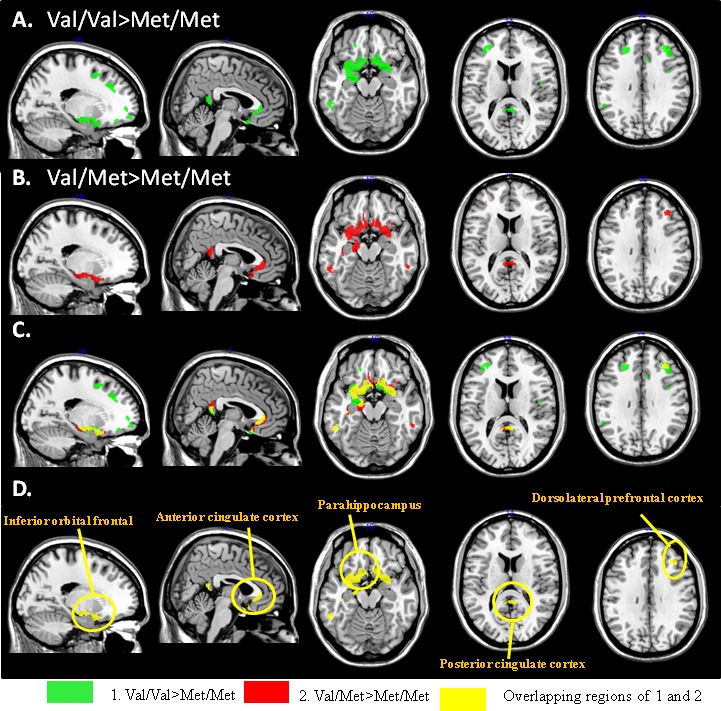
Peak clusters showing significant interactions of Val/Val > Met/Met (green) or Met/Val > Met/Met (red) from the entorhinal seed There were five peak clusters showing overlapping of Val/Val > Met/Val > Met/Met (yellow). (x,y,z) = Montreal Neurological Institute coordinates.

For each seed, we further explored the genotypic interactions with regards to the topography showing differences in structural covariance between seed and peak clusters (Figure 2 and Supplementary Figure 2).

Only peak clusters connected to the right entorhinal seed (Table 2) showed topographic similarities in structural covariance in genotype group analysis (Figure 2A: Val/Val > Met/Met green clusters; Figure 2B: Val/Met > Met/Met red clusters). The spatially coherent overlapping regions were located in the sub-regions of the prefrontal lobes and PCC (Figure 2C), of which the peak clusters sharing greater topographic distributions in Val/Val > Met/Met than Val/Met > Met/Met included the inferior orbital frontal, pregenual anterior cingulate cortex, para-hippocampus, PCC and dorsolateral prefrontal cortex (Figure 2D).

**Table 2:** Connectivity differences between brain-derived neurotrophic factor genotypes with right entorhinal cortex as seed

Main Cluster	Peak regions		Stereotaxic coordinates			Extent	Max T	*P*-value
		Side	x	y	z			
Val/Met>Met/Met								
Frontal inferio triangular region		R	38	35	24	357	3.69	<0.001
Inferior Temporal		L	−48	−25	−24	341	3.67	<0.001
Superior Temporal Pole		L	−27	8	−20	4931	3.61	<0.001
	Anteiror Cingulum	L	−6	38	−5	s.c.	3.5	<0.001
	Rectus	R	11	24	−17	s.c.	3.29	0.001
Precuneus		R	8	−45	15	820	3.3	0.001
	Posterior cingulum	L	−8	−46	16	s.c.	3.29	0.001
	Precuneus	L	−12	−52	21	s.c.	3.2	0.001
Inferior Temporal		L	−53	−46	−14	379	3.28	0.001
Middle Frontal		L	−35	38	18	143	3.27	0.001
Inferior Temporal		R	51	−24	−24	478	2.71	0.004
	Inferior Temporal	R	48	−33	−24	s.c.	2.53	0.006
	Inferior Temporal	R	56	−42	−15	s.c.	2.51	0.007
Val/Val>Met/Met								
Middle Frontal		L	−35	38	16	1315	4.5	<0.001
	Middle Frontal	L	−26	32	30	s.c.	3.3	0.001
	Middle orbital Frontal	L	−35	44	−9	s.c.	3.13	0.001
Frontal inferio triangular region		R	38	32	24	938	4.24	<0.001
	Middle Frontal	R	41	24	33	s.c.	2.92	0.002
	Middle Frontal	R	35	42	13	s.c.	2.65	0.005
Superior Temporal Pole		L	−27	8	−20	6427	4.02	<0.001
	Rectus	R	12	24	−15	s.c.	3.65	<0.001
	Anteiror Cingulum	R	3	29	−3	s.c.	3.59	<0.001
Supplementary Motor Area		L	−11	−12	54	812	3.74	<0.001
	Superior Frontal	L	−15	17	51	s.c.	3.17	0.001
	Superior Frontal	L	−17	8	48	s.c.	3.13	0.001
Precuneus		L	−12	−52	21	612	3.59	<0.001
	Posterior cingulum	R	3	−42	9	s.c.	3.02	0.002
	Precuneus	R	11	−46	16	s.c.	2.91	0.002
Insula		R	47	3	0	1495	3.53	<0.001
	Insula	R	41	−12	−3	s.c.	2.86	0.003
	Insula	R	39	−15	9	s.c.	2.64	0.005
Inferior Temporal		L	−47	−25	−24	1146	3.34	0.001
	Inferior Temporal	L	−53	−46	−15	s.c.	3.03	0.002
	Middle Temporal	L	−56	−43	−8	s.c.	2.81	0.003
SupraMarginal		L	−54	−46	28	123	3.01	0.002
Middle Temporal		R	48	−5	−27	171	2.98	0.002
	Inferior Temporal	R	50	−11	−33	s.c.	2.48	0.008
	Superior medial Frontal	L	−17	54	1	289	2.87	0.003
	Superior orbital frontal	L	−14	54	−11	s.c.	2.59	0.005
	Middle orbital Frontal	L	−24	53	−11	s.c.	2.57	0.006
Middle Temporal		L	−44	−63	21	115	2.73	0.004
	Insula	L	−35	18	0	188	2.55	0.006
	Frontal inferior operculum	L	−39	14	13	s.c.	2.37	0.01

Peak regions are within the Main cluster; R=right, L=left; s.c=same cluster

Max T is the maximum T statistic for each local maximum. *P* < 0.01 based on non-stationary cluster-extent False discovery rate correction.

For the PCC (Supplementary Figure 2A), frontoinsular (Supplementary Figure 2B) and dorsolateral prefrontal (Supplementary Figure 2C) seeds, differences in covariance between Val/Val > Met/Met or Val/Met > Met/Met were located in different sub-regions of the brain (Supplementary Tables 13-15). Of note, in the covariance linking the PCC, frontoinsular and dorsolateral prefrontal seeds, there were no overlapping regions of Val/Val > Val/Met > Met/Met, suggesting a lack of a genetic dosage effect from these three seeds.

### Dosage effects (Val/Val > Val/Met > Met/Met) of the BDNF val allele *via* entorhinal seed-based connectivity covariance strength alterations

**Figure 3:**
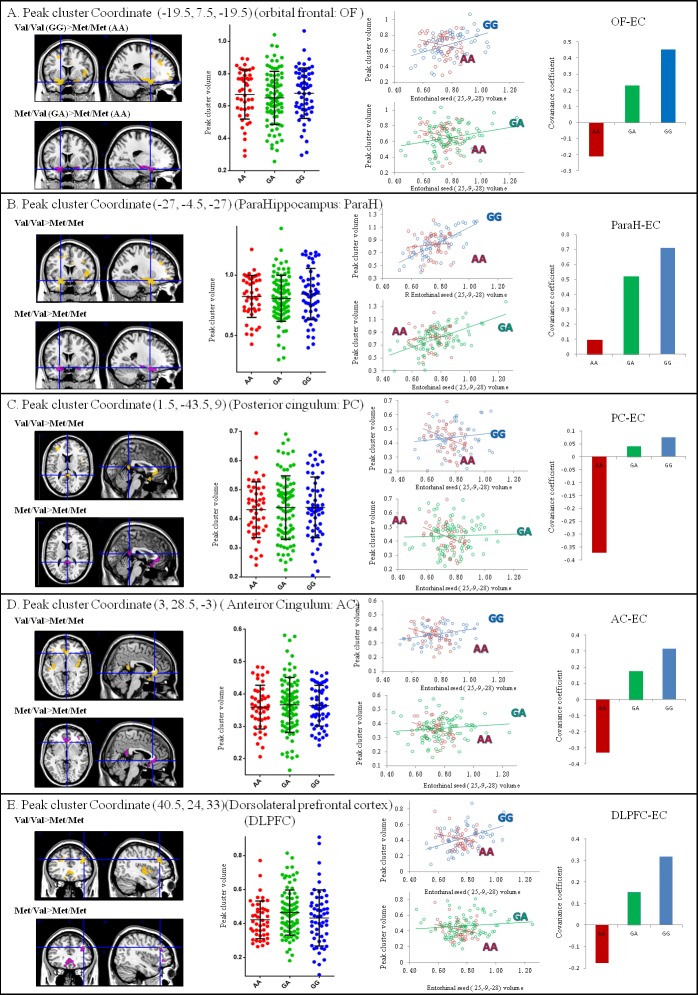
Correlation analysis between entorhinal seed volume and peak cluster volume showing significant interactions of the BDNF genotypes (*p* < 0.05) The gray matter volumes were extracted from a 4-mm radius sphere in the peak voxel expressing significance. Blue dots and lines represent Val/Val (G homozygotes). Green dots and lines represent Val/Met (GA allele). Red dots and lines represent Met/Met (A homozygotes). No significant changes were found in peak cluster volumes. (x,y,z) = Montreal Neurological Institute coordinates.

To investigate whether there was a genetic dosage effect, the volumes of the five peak clusters that were connected with the entorhinal seed were extracted, as they showed significant interactions between Val/Val > Met/Met or Val/Met > Met/Met (Figure 3). While the entorhinal seed volume (Figure 1B) and seed-connected cluster volume (Figure 3A-3E) were not significantly different among the three genotypes, the genetic dosage effect of the Val allele was *via* changes in the strength of entorhinal seed-based covariance connectivity (Val/Val > Val/Met > Met/Met) (Figure 3).

### Clinical significance of entorhinal seed-related peak clusters showing genotype differences

**Figure 4:**
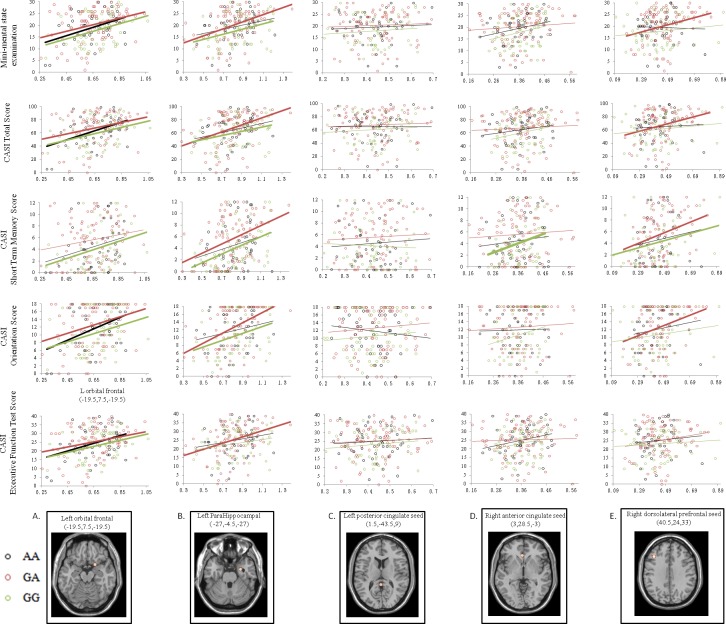
Correlation analysis between cognitive test scores and volume of the peak clusters of the default mode network medial temporal subsystem Green dots and lines represent Val/Val (G homozygotes). Red dots and lines represent Val/Met (GA allele). Black dots and lines represent Met/Met (A homozygotes). Correlations with statistical significance (*p* < 0.05) in each genotype group are highlighted with thicker lines. (x,y,z) = Montreal Neurological Institute coordinates.

The clinical significance of each peak cluster linking to the entorhinal seed showing a genotype effect was evaluated by correlation analysis (Figure 4). Among the five peak cluster volumes and in all three genotypes, only the left orbitofrontal cluster volume (Figure 4A, bold lines) demonstrated significant correlations in predicting MMSE, CASI total scores, CASI orientation and EFT scores.

## DISCUSSION

The results of this study provide data on the network-specific genetic influence of BDNF Val66Met in the early stages of AD. There were three main findings. First, the seed-based SCN pattern validated the hypothesis that the BDNF functional polymorphism targets large-scale brain networks rather than selectively in a focal brain region. Second, a genetic dosage effect of the BDNF Val allele was found only in the DMN medial temporal subsystem with cortical hubs consisting of entorhinal, PCC and prefrontal regions. The presence of the genetic dosage effect suggests that the mechanism related to this functional polymorphism may be *via* modulation of the strength of structural covariance between spatially scattered but functionally coherent regions rather than having a direct impact on the seed or peak cluster volume. Third, while the volumes of the seed and peak clusters of the DMN medial temporal subsystem correlated significantly with cognitive test scores, only the orbitofrontal peak cluster volume predicted cognitive test scores in the three BDNF genotype groups.

### Network-specific genetic influence on DMN medial temporal lobe subsystem

The salient episodic memory impairment in AD has been reported to be strongly associated with hippocampal/entorhinal volume [35, 36], while decreased expressions of BDNF and its receptor, tyrosine receptor kinase B, were also located in the hippocampal and frontal regions [37]. Pathological molecules of the amyloid or tau protein may spread *via* synaptic connections, and BDNF has been reported to attenuate the neurotoxicity [5, 8]. The correlation of structural covariance between regions could therefore reveal the genetic effect of AD at the network level. Taken together with our results, the clinical features, BDNF activity and hippocampal network appear to be highly correlated in the early stages of AD.

While our results suggest that there was no direct effect of BDNF genotype on hippocampal or entorhinal volume, we found that the BDNF genotype may specifically modulate the DMN medial temporal lobe subsystem structural covariance patterns, of which the increased correlations between entorhinal seed and peak clusters demonstrated the genetic dosage effect of the Val allele. While early amyloid burden or GM atrophy has been reported to involve the DMN medial temporal lobe subsystem in the early stage of AD [38], lower BDNF protein levels in the entorhinal cortex seed region have also been reported [39] implying the vulnerability of this region. Therefore, within the entorhinal seed and DMN, BDNF activity can be regarded as an important factor against pathological toxicity.

These alterations can be caused by several factors, and the disease- and genetic-related changes in structural covariance can be difficult to interpret. As the cortical hubs of the DMN are highly functionally anchored, the spatially scattered DMN-related clusters that show structural covariance may validate the notion that BDNF Val66Met polymorphisms only affect activity-dependent pathways [9]. Attenuated correlations between brain regions may suggest disconnectivity related to lower BDNF activity. The explanation of the dosage effect using correlation strength between seed and seed-based connectivity clusters was based on reports that neurons transfected with Met-BDNF show diminished neuronal integrity [9] and less hippocampal dendritic complexity in haploinsufficient BDNF mice [40]. In transgenic mice (BDNF [Met/Met]) [41], normal levels of BDNF have been reported in the brain despite defective secretion from neurons, suggesting the genetic susceptibility of Met homozygotes. As such, the seed region of Met carriers may show less connectivity in mediating the cortical hubs of the DMN network. Alternatively, localized or limited degeneration of a covariance network is also possible in patients with the Met allele [42].

In contrast, stronger correlations modulated by increased BDNF activity may suggest greater regional connectivity and synchronized GM loss in regions targeted by the pathological process. Although the pair-wise correlations did not necessarily influence the global organizational properties of structural covariance, as shown by the lack of differences in regional volumes among genotypes, the significant correlations between cognitive outcomes and seed (or peak clusters such as the anterior cingulate, orbitofrontal and dorsolateral prefrontal lobes) volumes within the DMN hubs still address the pathological-genetic alterations of local clustering. The findings of the DMN still reflect segregated and less integrated components in AD networks that could predict cognitive outcomes.

### BDNF genotype polymorphisms target different GM degenerative network patterns

Among the four seed-based SCNs, the Val (Val/Val and Val/Met) carriers had more extended voxels of structural covariance in the DMN medial temporal lobe subsystem. In contrast, the Met (Met/Met and Val/Met) carriers showed stronger structural associations in the DMN midline core system, salience and executive control networks. As BDNF activity was different among the genotypes, the SCN patterns may reflect different strengths of structural covariance between cortical GM and seed region. The SCN patterns may therefore provide evidence that BDNF genotypes target specific large-scale brain networks in patients with AD that may modulate distinct degenerative patterns. In normal human cortical morphology, Met carriers have been reported to have smaller hippocampal and prefrontal cortical volumes [12, 43]. However, the genetic effects of Met carriers on smaller entorhinal or prefrontal cortical volumes were not established in this study.

Studies on the genetic risk of developing AD [10, 11] or the direct influence of BDNF Val66Met functional polymorphisms on regional volume have reported inconsistent results [12–15, 44]. As significant apolipoprotein E and BDNF interactions have been reported in normal elderly and AD patients to predict episodic memory performance [45–48], our study design may ignore the epistatic effect of the apolipoprotein E gene. However, linkage disequilibrium either from other functional polymorphisms in the BDNF gene or from another nearby gene was still possible. Taken together, the strength of connectivity alterations with regards to the interactions of genotypes supports the hypothesis that BDNF functional activity contributes to cognitive outcomes in the early stages of AD, and that this may be mediated by polymorphisms at the Val66Met locus.

Possible explanations why the BDNF genotypes showed inconsistent results on brain structures may be related to epistasis [44], variability in individual hippocampal activity and its neural substrates [49], differences in racial distribution of the Met allele with differences in group strategies for BDNF genotypes [50], and interactions between genotypes and specific intracerebral pathology [51]. In surgically-resected hippocampi from patients with epilepsy, increased levels of BDNF mRNA and protein have been noted, indicating that epileptic activity may up-regulate protein levels *via* BDNF gene expression [52, 53]. In patients with chronic epilepsy, however, decreased BDNF secretion has been associated with a decompensated hippocampus [54].As such, secretion of BDNF can be dynamic depending on the integrity of the hippocampus, disease typs and thus the disease stage. We enrolled AD patients with an early stage of disease and the network influence could be different at different stages of disease.

### GM alterations reflected the endophenotype of the BDNF genotype

Despite our observations that BDNF Val66Met polymorphisms may affect structural covariance patterns, there were no significant differences in cognitive measurements in our genotype groups. On the basis of the none-significance in the PCC and dorsolateral prefrontal seed volumes among the three genotype groups, the volumes of both seeds were still significantly correlated with the cognitive scores. The finding supports the hypothesis that brain structure may be considered to be an endophenotype that is more sensitive to a genetic effect than behavioral level. The PCC is an early target of amyloid deposition in both patients with AD [55] and in cognitively normal subjects with a positive family history of AD [56]. The dorsolateral prefrontal cortex has been reported to frequently confer executive function, and activation of the prefrontal neural resources have been reported to compensate for the posterior degenerative process [57].

### Study limitations

An important limitation of this study is that we did not include a control group. The effects of BDNF Val66Met genetic variations on regional GM volume and functional state in healthy subjects have been reported [16, 47, 50]. The enrolment of controls may help to understand how the disease interacts with degenerative processes, and whether different genotypes have similar effects on normative structures. Nonetheless, the aim of this study was to determine whether changes in structural correlations between areas of the brain are AD-specific. Our results may provide further evidence reported in healthy elders, that genetic variations of BDNF in AD may mediate SCN patterns and the strength of structural covariance rather than focally. Another potential limitation is that we used seed-based analysis, with an emphasis on the SCNs and reported the genetic dosage effect focusing on the Val allele. The use of independent component analysis [58] or resting state functional MRI data may elucidate whether other networks are involved and also validate the findings of the DMN medial temporal network observed in this study. Finally, as the clinical significance was established in restricted nodes showing peak correlation, whether these nodes were affected by other genetic or biological effects remains an important issue that needs to be tested in further studies. Nonetheless, our genotype groups were carefully matched in major possible confounders, highlighting the unique role of this BDNF functional polymorphism.

## CONCLUSIONS

In summary, our structural covariance analysis supports the role of BDNF functional polymorphisms in modulating the GM degenerative scaffold in the early stages of AD. Our results suggest that the Val66Met functional polymorphism carries different weightings on the DMN, salience and executive control networks. Within the DMN medial temporal lobe subsystem, the genetic dosage effect of the Val allele on entorhinal seed-based SCN was validated by correlation strength gradients between seed and peak clusters, thus supporting the importance of BDNF in AD degenerative network.

## MATERIALS AND METHODS

This study was conducted in accordance with the Declaration of Helsinki and was approved by the Institutional Review Board of Chang Gung Memorial Hospital. The study participants were treated at the Cognition and Aging Center, Department of General Neurology, Kaohsiung Chang Gung Memorial Hospital. A total of 186 subjects (82 males, 104 females) were included after the consensus of a panel composed of neurologists, neuropsychologists, neuroradiologists and experts in nuclear medicine [59]. AD was diagnosed according to the International Working Group criteria [60] with a clinical diagnosis of typical AD. All of the patients had a clinical dementia rating scores of 0.5 or 1, and all of the patients were in a stable condition under treatment with acetylcholine esterase inhibitors from the time of diagnosis. The exclusion criteria were a history of clinical stroke, a modified Hachinski ischemic score > 4 [61] and depression.

### Study working scheme

The patients were classified into three groups based on the genotype: Met/Met homozygotes (*n* = 45), Met/Val (*n* = 86) and Val/Val homozygotes (*n* = 55). To avoid possible confounding, the three genotype groups were matched for gender, age, educational level, clinical dementia rating [62] and Mini-Mental State Examination (MMSE) scores [63] (Table 1). The working scheme was as follows. First, the SCN was established by seed-based correlation analysis. Seed regions anchoring the DMN (medial temporal subsystem or midline core subsystem), salience and executive control networks were selected to generate SCNs. Differences in each seed regional volume and SCN peak cluster volume were then compared among the three genotype groups. Finally, to evaluate the genetic dosage effects of the Val allele on SCN clusters [9], the SCNs showing significant genotype interactions (i.e. Val/Val > Met/Met or Met/Val > Met/Met) in seed-peak cluster covariance were modeled. Correlations between peak clusters showing a dosage effect with cognitive test scores were assessed to evaluate the clinical significance.

### Clinical and neurobehavioral assessments

After enrolment, demographic data and a family history of each patient were recorded, and each patient underwent physical and neurological examinations. A trained neuro-psychologist administered the tests. The 30-item MMSE [63] and cognitive ability-screening instrument (CASI) [64] total scores were used as a global assessment of cognitive function. Attention, verbal fluency, abstract thinking, and mental manipulation sub-domain scores of the CASI were used to assess executive function test (EFT) [65], while the non-executive domains included orientation, short- and long-term memory, language ability, and drawing. We used the neuropsychiatric inventory (NPI) to evaluate changes in behavior.

### Genotyping

Genomic DNA was extracted from blood samples using a commercial kit (Qiagen, Gentra Puregene Blood Kit), followed by genotyping for BDNF Val66Met polymorphisms using the polymerase chain reaction (PCR)-restriction fragment length polymorphism method. Genotyping was conducted with the operator blinded to the clinical data. The apolipoprotein E4 genotype was also determined using a PCR-restriction fragment length polymorphism assay and restriction enzyme HhaI [66]. Apolipoprotein E4 carriers were defined as those with one or two E4 alleles [59].

### Cerebrovascular risk confounders

It has been reported that factors such as oxidative stress, deregulated neuroinflammation and an elevated blood sugar level are related to changes in BDNF expression [67–69]. We included the following cerebrovascular risk confounders: age, homocysteine, total cholesterol, triglycerol, high-density lipoprotein (HDL), low-density lipoprotein (LDL), creatinine, hemoglobin, vitamin B12, folate, and hemoglobin-A1C [36].

### Image acquisition

MR images were acquired using a 3.0T MRI scanner (Excite, GE Medical Systems, Milwaukee, WI, USA). Structural images were acquired for structural covariance analysis using the following protocols: a T1-weighted, inversion-recovery-prepared, three-dimensional, gradient-recalled acquisition in a steady-state sequence with a repetition time/echo time/inversion time of 8,600 ms/minimal/450 ms, a 256 × 256 mm field of view, and a 1-mm slice sagittal thickness with a resolution of 0.5 × 0.5 × 1 mm^3^.

### Data analysis

Image preprocessing and statistical analysis were performed using SPM8 (SPM8, Wellcome Trust Centre of Cognitive Neurology, University College London, UK, http://www.fil.ion.ucl.ac.uk/spm/). The T1 images were reoriented, realigned, and normalized using the standard Montreal Neurological Institute (MNI) space. The images were then segmented into GM and white matter. Related tissue segments were used to create a custom template using the diffeomorphic anatomical registration using exponentiated lie algebra (DARTEL) approach. The DARTEL approach is one of the highest ranking registration methods in patients with AD [70]. The modulated and warped images were then smoothed using a Gaussian kernel of 8 mm full width at half maximum. A direct comparison among the modulated segmented GM volumes of Val/Val homozygotes, Met/Val and Met/Met homozygotes using voxel-based morphometry [71] showed no significant differences with the threshold set at *p* < 0.05, corrected for a false discovery rate (FDR) with a cluster size > 100 voxels.

### Statistic analysis

Clinical and laboratory data were expressed as mean ± standard deviation. Analysis of variance with Bonferroni correction for multiple comparisons was used to compare levels of cerebrovascular risk biomarkers or continuous variables among the Val/Val homozygotes, and Met/Val and Met/Met homozygotes. All statistical analyses were conducted using SPSS software (SPSS version 22 for Windows^®^, SPSS Inc., Chicago, IL). Statistical significance was set at *p* < 0.05.

To investigate the SCNs, the regional GM volumes of four regions of interest (ROIs) were extracted from the 186 preprocessed images. The seed ROI included the right entorhinal cortex (coordinates: 25,−9,−28), left posterior cingulate cortex (PCC; coordinates: −2,−36, 35), right frontoinsular cortex (coordinates: 38, 26,−10), and right dorsolateral prefrontal cortex (coordinates: 44, 36, 20) (Figure 1A). According to the literature, these regions anchor the DMN medial temporal subsystem (right entorhinal cortex) [72], DMN midline core subsystem (left PCC) [73, 74], salience network (right frontoinsular cortex), and executive network (right dorsolateral prefrontal cortex) [23]. As the pathology or functional connectivity in typical patients with AD is distributed symmetrically, we did not perform a contralateral seed analysis in this study.

From the modified GM images, the GM volumes of a 4-mm radius sphere around the seed ROI coordinates were calculated, followed by four separate correlation analyses using the extracted GM volumes as the covariates of interest. The three BDNF genotype groups were modeled separately. For each BDNF genotype group, specific contrasts were set to identify voxels that showed positive correlations for each seed ROI. The results reflected the SCNs of each ROI and the threshold was set at *p* < 0.01, corrected for FDR with a cluster size > 100 voxels.

In addition, to investigate how genetic variance may interfere with structural covariance patterns, voxels showing significant differences in the regression slopes in each seed-peak cluster correlations were compared, pointing to possible interactions between Val/Val > Met/Met or Val/Met > Met/Met. The genetic dosage model was based on an *in vivo* study in which the expression of BDNF was found to be highest in Val/Val, followed by Val/Met and Met/Met [75]. Specific t contrasts were established to map the voxels that expressed significant between-group associations. The threshold for the resulting statistical parametric maps was set at *p* < 0.001 (uncorrected) with a cluster size > 100 voxels. In addition, for the peak clusters showing significant between-group differences, a 4-mm radius sphere was placed on the peak voxel, and the GM volumes were then calculated for regression analysis. The seed ROI was considered to be the predictive variable for the extracted SCN peak voxel, and the threshold was set at *p* < 0.05 with multiple corrections. To evaluate the clinical significance of the seed or identified peak voxel, we used a linear regression model with the cognitive test scores serving as the dependent variable. The threshold was set at *p* < 0.05 with multiple corrections.

## SUPPLEMENTARY MATERIALS


