# Supplementary material for: Dose-dependent genotype effects of BDNF Val66Met polymorphism on default mode network in early stage Alzheimer's disease
Source: Oncotarget. 2016 Aug 2;7(34):54200–14. doi: 10.18632/oncotarget.11027 (PMC5342335; doi:10.18632/oncotarget.11027)
Supplement: Supplementary file 1 [file oncotarget-07-54200-s001.pdf]

Dose-dependent genotype effects of BDNF Val66Met polymorphism on default mode network in early stage Alzheimer’s disease

Supplementary Materials

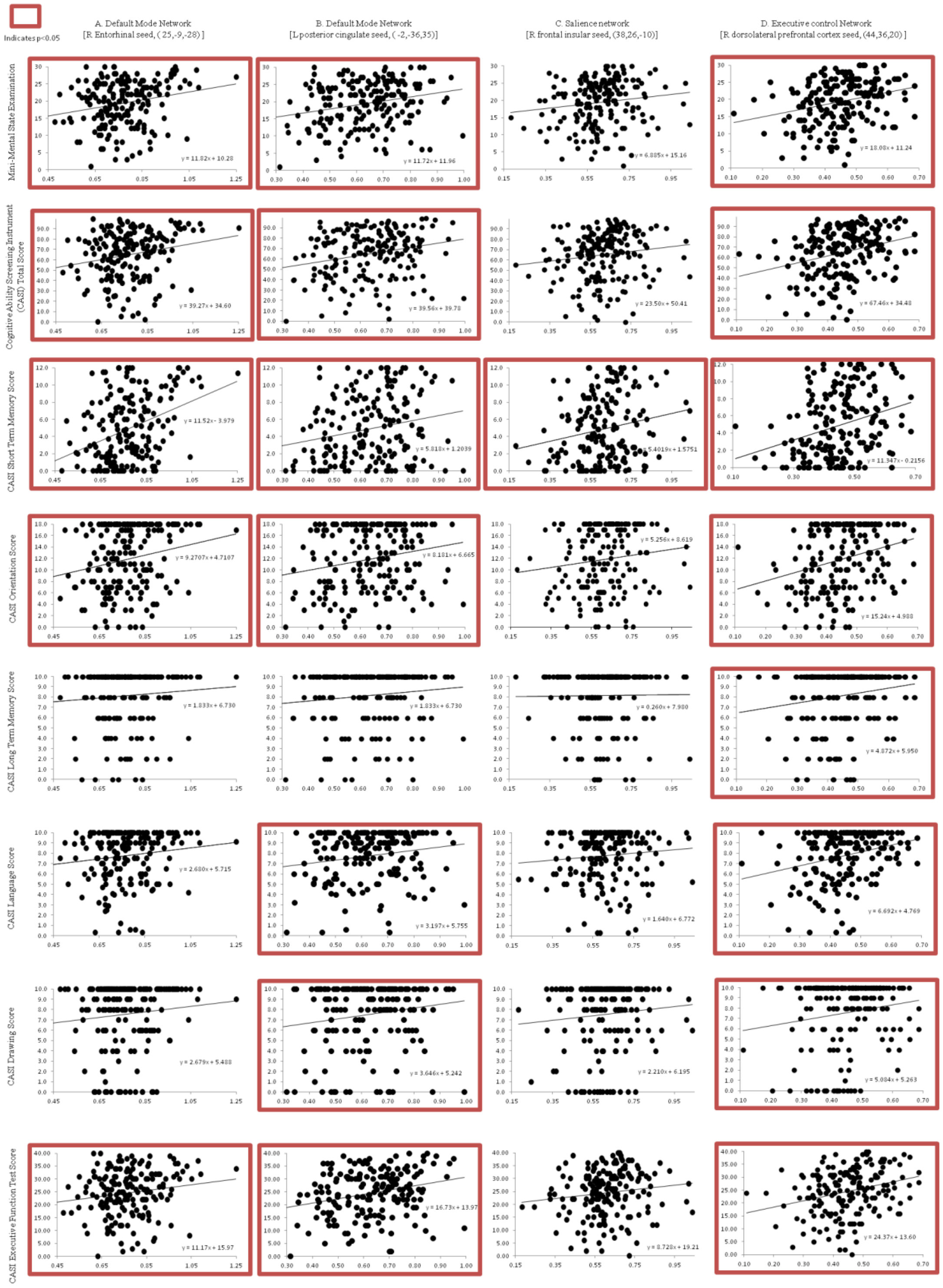

Supplementary Figure 1. Correlations and regression analysis between the clinical

scores and gray matter (GM) volumes extracted from a 4-mm radius sphere centered on the seed region of each network. Correlation analysis showing statistical significance ($p<0.05$) is highlighted within the red box. The X axis represents the volume of each seed. (x,y,z) = Montreal Neurological Institute coordinates.

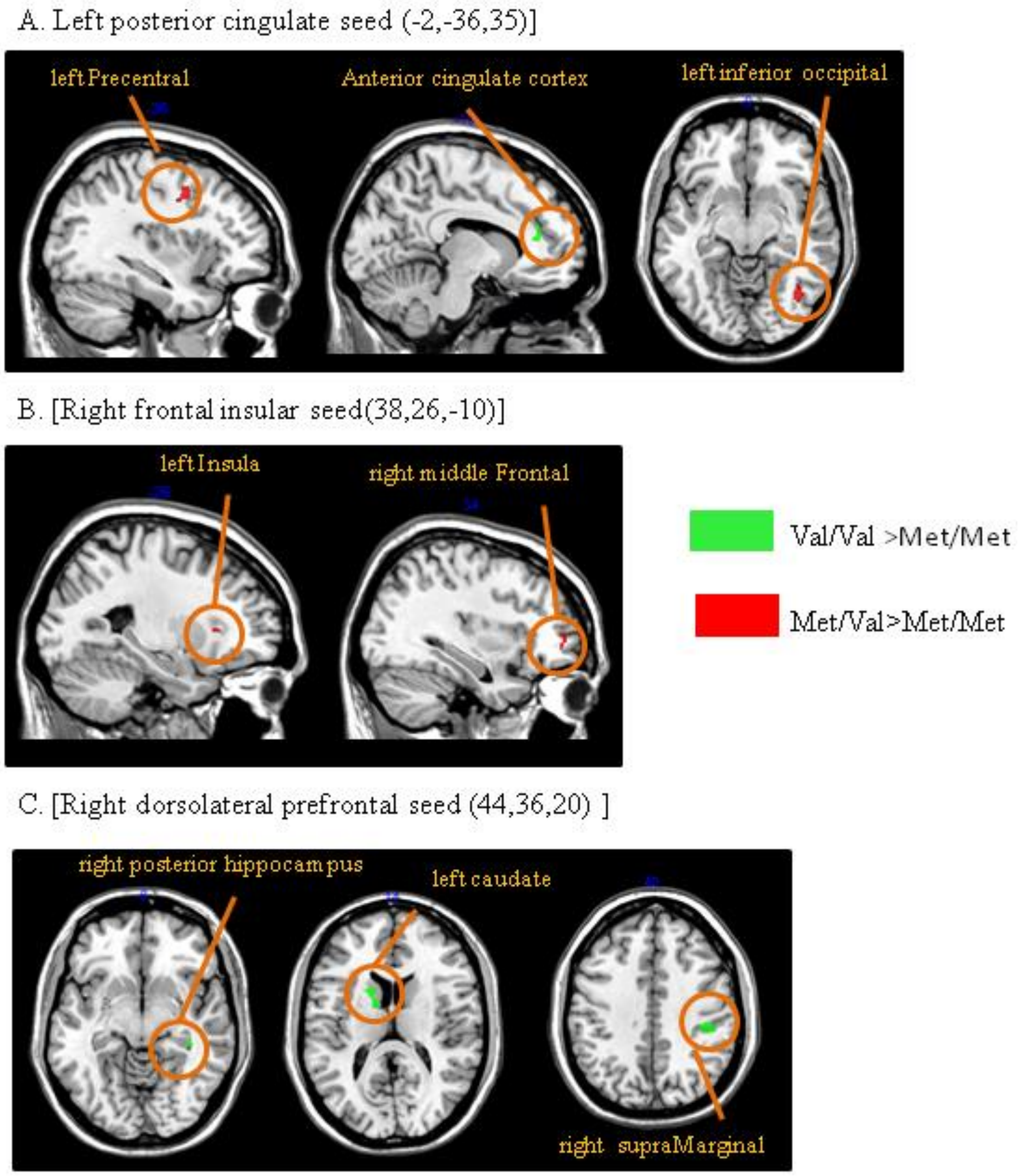

Supplementary Figure 2. Peak clusters showing significant interactions of Val/Val >

Met/Met (green) or Met/Val > Met/Met (red) from the (A) left posterior cingulate seed, (B) right frontal insular seed, and (C) right dorsolateral prefrontal seed. (x,y,z) = Montreal Neurological Institute coordinates.

Supplementary Table 1. Structural covariance network for brain-derived neurotrophic factor Met/Met with right entorhinal cortex as seed.

**Main Cluster**	**Peak regions**	**Stereotaxic coordinates**				**Extent**	**Max T**	**P-value**
		Side	x	y	z			
**No significant clusters exceed the threshold**								

Peak regions are within the Main cluster.
Max T is the maximum T statistic for each local maximum. P<0.01 based on non-stationary cluster-extent False discovery rate correction.

Supplementary Table 2. Structural covariance network for brain-derived neurotrophic factor Met/Val with right entorhinal cortex as seed.

Main Cluster	Peak regions		Stereotaxic coordinates			Extent	Max T	P-value
		Side	x	y	z			
**ParaHippocampal**		**R**	**27**	**-10**	**-29**	**46915**	**24.06**	**<0.001**
	Fusiform	R	33	-1	-41	s.c.	8.44	<0.001
	ParaHippocampal	L	-20	-9	-26	s.c.	7.72	<0.001
**Supp_Motor_Area**		**L**	**-14**	**-10**	**66**	**760**	**4.76**	**<0.001**
	Superior Frontal	L	-14	-3	72	s.c.	4.37	<0.001
	Precentral	L	-26	-16	64	s.c.	3.24	0.001
**Cerebelum_8**		**R**	**29**	**-51**	**-45**	**161**	**4.32**	**<0.001**
**Superior Frontal**		**R**	**27**	**-9**	**63**	**429**	**4.21**	**<0.001**
	Superior Frontal	R	23	6	58	s.c.	3.8	<0.001
	Superior Frontal	R	20	14	64	s.c.	3.22	0.001
**SupraMarginal**		**L**	**-60**	**-24**	**31**	**191**	**4.11**	**<0.001**
	SupraMarginal	L	-60	-37	33	s.c.	3.43	<0.001
**Cerebelum_Crus1**		**L**	**-23**	**-70**	**-33**	**654**	**4.09**	**<0.001**
	Cerebelum_Crus1	L	-14	-72	-30	s.c.	3.99	<0.001
**Middle Frontal**		**R**	**26**	**39**	**37**	**140**	**3.9**	**<0.001**
**Middle Occipital**		**R**	**38**	**-73**	**25**	**1358**	**3.9**	**<0.001**
	Angular	R	38	-64	42	s.c.	3.88	<0.001
	Angular	R	39	-72	36	s.c.	3.72	<0.001
**Middle orbital Frontal**		**R**	**35**	**57**	**-3**	**236**	**3.89**	**<0.001**
**Superior Frontal**		**R**	**18**	**56**	**27**	**237**	**3.88**	**<0.001**
	Superior medial Frontal	R	5	59	10	s.c.	3.2	0.001
**Inferior orbital Frontal**		**L**	**-33**	**26**	**-15**	**623**	**3.81**	**<0.001**
	Inferior orbital Frontal	L	-23	23	-17	s.c.	3.75	<0.001
	Inferior orbital Frontal	L	-20	26	-26	s.c.	3.62	<0.001
**Middle Cingulum**		**L**	**-8**	**-25**	**33**	**348**	**3.73**	**<0.001**
**SupraMarginal**		**R**	**62**	**-45**	**31**	**260**	**3.67**	**<0.001**
	SupraMarginal	R	65	-34	39	s.c.	3.66	<0.001
**Cerebelum_6**		**R**	**18**	**-63**	**-26**	**104**	**3.63**	**<0.001**

**Frontal inferior operculum**		**R**	**53**	**9**	**28**	**304**	**3.59**	**<0.001**
	Precentral	R	60	2	31	s.c.	3.42	<0.001
	Frontal inferior operculum	R	45	12	34	s.c.	3.31	0.001
**Precuneus**		**L**	**-17**	**-52**	**67**	**186**	**3.55**	**<0.001**
	Superior Parietal	L	-21	-61	61	s.c.	3.34	0.001
**Middle Frontal**		**R**	**30**	**53**	**15**	**179**	**3.46**	**<0.001**
	Superior Frontal	R	21	53	16	s.c.	3.35	0.001
**Middle Occipital**		**L**	**-41**	**-73**	**33**	**157**	**3.41**	**0.001**
**Middle Cingulum**		**L**	**-8**	**3**	**39**	**124**	**3.38**	**0.001**
	Middle Cingulum	L	-8	8	31	s.c.	3.25	0.001
	Middle Cingulum	L	-8	-3	33	s.c.	3.23	0.001

Peak regions are within the Main cluster; R=right, L=left, s.c= same cluster
Max T is the maximum T statistic for each local maximum. P<0.01 based on non-stationary cluster-extent False discovery rate correction.

Supplementary Table 3. Structural covariance network for brain-derived neurotrophic factor Val/Val homozygotes with right entorhinal cortex as seed.

Main Cluster	Peak regions		Stereotaxic coordinates			Extent	Max T	P-value
		Side	x	y	z			
**ParaHippocampal**		**R**	**27**	**-10**	**-29**	**23751**	**15.76**	**<0.001**
	Inferior Temporal	R	35	0	-42	s.c.	8.17	<0.001
	Inferior Temporal	R	45	6	-42	s.c.	7.13	<0.001
**ParaHippocampal**		**L**	**-29**	**-4**	**-27**	**18372**	**7.56**	**<0.001**
	ParaHippocampal	L	-27	-15	-27	s.c.	7.44	<0.001
	Inferior Temporal	L	-32	5	-36	s.c.	6.8	<0.001
**Superior Frontal**		**R**	**27**	**-7**	**58**	**708**	**5.12**	**<0.001**
**Superior orbital frontal**		**L**	**-17**	**56**	**-12**	**2850**	**5.07**	**<0.001**
	Middle Frontal	L	-35	39	16	s.c.	4.88	<0.001
	Middle Frontal	L	-30	47	10	s.c.	4.65	<0.001
**Frontal inferio triangular region**		**L**	**-41**	**33**	**3**	**145**	**5.05**	**<0.001**
**Middle Frontal**		**R**	**38**	**33**	**39**	**1611**	**4.89**	**<0.001**
	Middle Frontal	R	30	21	49	s.c.	4.4	<0.001
	Middle Frontal	R	29	42	34	s.c.	4.07	<0.001
**Angular**		**R**	**36**	**-63**	**40**	**196**	**4.3**	**<0.001**
	Middle Occipital	R	38	-69	33	s.c.	3.47	0.001
**Middle orbital Frontal**		**R**	**35**	**53**	**-15**	**393**	**4.18**	**<0.001**
	Middle orbital Frontal	R	27	62	-11	s.c.	4.03	<0.001
	Superior orbital frontal	R	15	56	-12	s.c.	3.65	<0.001
**Middle Occipital**		**R**	**36**	**-75**	**18**	**134**	**4.16**	**<0.001**
**Precentral**		**L**	**-32**	**-7**	**55**	**108**	**4.02**	**<0.001**
**Superior medial Frontal**		**L**	**-5**	**60**	**4**	**284**	**3.75**	**<0.001**
	Anteiror Cingulum	**L**	**-9**	**48**	**9**	**s.c.**	**3.39**	**0.001**
	Anteiror Cingulum	L	-9	41	0	s.c.	3.29	0.001
**Superior Frontal**		**R**	**23**	**59**	**4**	**221**	**3.66**	**<0.001**
	Superior medial Frontal	R	11	57	12	s.c.	3.45	0.001
**Cuneus**		**L**	**-11**	**-73**	**27**	**171**	**3.55**	**<0.001**

Peak regions are within the Main cluster; R=right, L=left, s.c= same cluster
Max T is the maximum T statistic for each local maximum. P<0.01 based on non-stationary cluster-extent False discovery rate correction.

Supplementary Table 4. Structural covariance network for brain-derived neurotrophic factor Met/Met homozygotes with left posterior cingulate as seed.

**Main Cluster**	**Peak regions**	**Stereotaxic coordinates**				**Extent**	**Max T**	**P-value**
		Side	x	y	z			
**Middle Cingulum**		**L**	**-2**	**-36**	**34**	**166885**	**28.47**	**<0.001**
	Middle Cingulum	L	-5	-21	46	s.c.	9.2	<0.001
	Middle Cingulum	L	-3	-7	40	s.c.	8.55	<0.001
**Vermis**			**0**	**-66**	**-41**	**649**	**3.73**	**<0.001**
	Cerebelum_Crus2	L	-8	-87	-41	s.c.	3.05	0.002

Peak regions are within the Main cluster; R=right, L=left, s.c= same cluster
Max T is the maximum T statistic for each local maximum. P<0.01 based on non-stationary cluster-extent False discovery rate correction.

Supplementary Table 5. Structural covariance network for brain-derived neurotrophic factor Met/Val allele with left posterior cingulate as seed.

Main Cluster	Peak regions	Stereotaxic coordinates				Extent	Max T	P-value
		Side	x	y	z			
**Middle Cingulum**		**L**	**-2**	**-36**	**34**	**75090**	**33.02**	**<0.001**
	Middle Cingulum	R	0	-7	42	s.c.	8.87	<0.001
	Superior medial Frontal	L	-2	26	51	s.c.	6.43	<0.001
**Thalamus**		**L**	**-5**	**-10**	**3**	**2931**	**5.05**	**<0.001**
**Postcentral**		**R**	**48**	**-25**	**45**	**1595**	**4.46**	**<0.001**
	SupraMarginal	R	35	-40	43	s.c.	4	<0.001
**Angular**		**R**	**53**	**-55**	**25**	**235**	**3.82**	**<0.001**
**Superior Frontal**		**L**	**-18**	**32**	**34**	**380**	**3.69**	**<0.001**
	Middle Frontal	L	-23	38	27	s.c.	3.69	<0.001
**Vermis**			**0**	**-46**	**-14**	**254**	**3.61**	**<0.001**
**Middle orbital Frontal**		**R**	**29**	**48**	**-11**	**200**	**3.59**	**<0.001**

Peak regions are within the Main cluster; R=right, L=left, s.c= same cluster
Max T is the maximum T statistic for each local maximum. P<0.01 based on non-stationary cluster-extent False discovery rate correction.

Supplementary Table 6. Structural covariance network for brain-derived neurotrophic factor Val/Val homozygotes with left posterior cingulate as seed.

Main Cluster	Peak regions	Stereotaxic coordinates				Extent	Max T	P-value
		Side	x	y	z			
**Middle Cingulum**		**L**	**-2**	**-36**	**34**	**20108**	**25.49**	**<0.001**
	Middle Cingulum	L	-2	-9	43	s.c.	6.64	<0.001
	Anteiror Cingulum	L	-6	42	13	s.c.	6.21	<0.001
**Angular**		**L**	**-48**	**-66**	**25**	**1465**	**5.61**	**<0.001**
	Inferior Parietal	L	-57	-54	37	s.c.	4.07	<0.001
	SupraMarginal	L	-53	-34	34	s.c.	3.97	<0.001
**SupraMarginal**		**R**	**63**	**-22**	**19**	**2187**	**5.51**	**<0.001**
	Postcentral	R	63	-15	25	s.c.	5.28	<0.001
	Rolandic Operculum	R	65	-15	12	s.c.	4.93	<0.001
**Inferior orbital Frontal**		**R**	**41**	**33**	**-17**	**388**	**4.53**	**<0.001**
**Superior Temporal Pole**		**L**	**-44**	**23**	**-17**	**879**	**4.45**	**<0.001**
	Inferior orbital Frontal	L	-38	32	-20	s.c.	4.24	<0.001
	Insula	L	-35	21	1	s.c.	4.09	<0.001
**Frontal inferio triangular region**		**L**	**-38**	**17**	**28**	**244**	**4.41**	**<0.001**
	Frontal inferior operculum	L	-39	12	21	s.c.	4.03	<0.001
**Superior Temporal**		**R**	**63**	**-13**	**-6**	**272**	**4.21**	**<0.001**
**Superior Temporal Pole**		**L**	**-54**	**12**	**-20**	**197**	**4.11**	**<0.001**
**Middle Temporal**		**L**	**-54**	**-39**	**-9**	**163**	**4.05**	**<0.001**
**Cerebelum_6**		**R**	**23**	**-58**	**-15**	**284**	**3.99**	**<0.001**
**Frontal inferio triangular region**		**R**	**35**	**24**	**12**	**150**	**3.77**	**<0.001**
	Frontal inferior operculum	R	41	17	6	s.c.	3.59	<0.001
**Middle Temporal**		**R**	**51**	**-63**	**4**	**151**	**3.63**	**<0.001**
	Inferior Temporal	R	54	-61	-3	s.c.	3.53	<0.001

Peak regions are within the Main cluster; R=right, L=left, s.c= same cluster
Max T is the maximum T statistic for each local maximum. P<0.01 based on non-stationary cluster-extent False discovery rate correction.

Supplementary Table 7. Structural covariance network for brain-derived neurotrophic factor Met/Met homozygotes with right frontoinsular cortex as seed.

Main Cluster	Peak regions	Stereotaxic coordinates				Extent	Max T	P-value
		Side	x	y	z			
**Inferior orbital Frontal**		**R**	**38**	**27**	**-11**	**20946**	**16.83**	**<0.001**
	ParaHippocampal	R	29	6	-26	s.c.	5.52	<0.001
	Middle Temporal Pole	R	33	9	-36	s.c.	5.33	<0.001
**Middle Temporal**		**L**	**-56**	**-45**	**10**	**16646**	**5.59**	**<0.001**
	Inferior orbital Frontal	L	-38	44	-12	s.c.	5.34	<0.001
	Inferior orbital Frontal	L	-35	35	-20	s.c.	5.31	<0.001
**Frontal inferior operculum**		**R**	**42**	**14**	**33**	**2620**	**5.18**	**<0.001**
	Precentral	R	54	-1	25	s.c.	4.96	<0.001
	Precentral	R	48	-7	37	s.c.	4.48	<0.001
**Superior Frontal**		**L**	**-23**	**5**	**58**	**860**	**4.83**	**<0.001**
	Precentral	L	-32	-13	42	s.c.	4.15	<0.001
**Superior medial Frontal**		**L**	**-12**	**59**	**19**	**716**	**4.53**	**<0.001**
	Superior Frontal	L	-14	50	36	s.c.	4.4	<0.001
	Superior Frontal	L	-26	54	22	s.c.	3.68	<0.001
**Precuneus**		**R**	**17**	**-67**	**46**	**695**	**4.28**	**<0.001**
	Angular	R	35	-64	42	s.c.	3.88	<0.001
	Angular	R	44	-67	30	s.c.	3.68	<0.001
**Lingual**		**R**	**26**	**-57**	**-6**	**605**	**4.24**	**<0.001**
**Superior Frontal**		**R**	**23**	**14**	**52**	**227**	**4.22**	**<0.001**
**Insula**		**R**	**39**	**-7**	**12**	**690**	**4.11**	**<0.001**
	Insula	R	45	-6	4	s.c.	4.1	<0.001
	Insula	R	42	8	4	s.c.	3.29	0.001
**Inferior Parietal**		**L**	**-42**	**-36**	**43**	**747**	**4.08**	**<0.001**
	Superior Parietal	L	-27	-61	43	s.c.	4.08	<0.001
	Inferior Parietal	L	-45	-46	36	s.c.	3.66	<0.001
**Middle Cingulum**		**R**	**9**	**15**	**43**	**612**	**4.03**	**<0.001**
	Anteiror Cingulum	R	12	35	25	s.c.	3.89	<0.001
	Middle Cingulum	R	9	23	33	s.c.	3.62	<0.001
**Middle Cingulum**		**R**	**8**	**-28**	**34**	**428**	**3.98**	**<0.001**

	Middle Cingulum	R	8	-28	43	s.c.	3.54	<0.001
	Middle Cingulum	R	8	-13	33	s.c.	3.4	0.001
**Cuneus**		**L**	**-5**	**-94**	**18**	**158**	**3.92**	**<0.001**
**Middle Cingulum**		**L**	**-12**	**-6**	**46**	**241**	**3.77**	**<0.001**
	Middle Cingulum	L	-6	-3	33	s.c.	3.73	<0.001

Peak regions are within the Main cluster; R=right, L=left, s.c= same cluster
Max T is the maximum T statistic for each local maximum. P<0.01 based on non-stationary cluster-extent False discovery rate correction.

Supplementary Table 8. Structural covariance network for brain-derived neurotrophic factor Met/Val allele with right frontoinsularcortex as seed.

Main Cluster	Peak regions	Stereotaxic coordinates				Extent	Max T	P-value
		Side	x	y	z			
**Middle Cingulum**		**L**	**-2**	**-36**	**34**	**75090**	**33.02**	**<0.001**
	Middle Cingulum	L	0	-7	42	s.c.	8.87	<0.001
	Superior medial Frontal	L	-2	26	51	s.c.	6.43	<0.001
**Thalamus**		**L**	**-5**	**-10**	**3**	**2931**	**5.05**	**<0.001**
**Postcentral**		**R**	**48**	**-25**	**45**	**1595**	**4.46**	**<0.001**
	SupraMarginal	R	35	-40	43	s.c.	4	<0.001
**Angular**		**R**	**53**	**-55**	**25**	**235**	**3.82**	**<0.001**
**Superior Frontal**		**L**	**-18**	**32**	**34**	**380**	**3.69**	**<0.001**
	Middle Frontal	L	-23	38	27	s.c.	3.69	<0.001
**Vermis_3**		**R**	**0**	**-46**	**-14**	**254**	**3.61**	**<0.001**
**Middle orbital Frontal**		**R**	**29**	**48**	**-11**	**200**	**3.59**	**<0.001**

Peak regions are within the Main cluster; R=right, L=left, s.c= same cluster
Max T is the maximum T statistic for each local maximum. P<0.01 based on non-stationary cluster-extent False discovery rate correction.

Supplementary Table 9. Structural covariance network for brain-derived neurotrophic factor Val/Val homozygotes with right frontoinsular cortex as seed.

Main Cluster	Peak regions	Stereotaxic coordinates				Extent	Max T	P-value
		Side	x	y	z			
**Inferior orbital Frontal**		**R**	**36**	**27**	**-11**	**1142**	**19.04**	**<0.001**

Peak regions are within the Main cluster; R=right, L=left, s.c= same cluster
Max T is the maximum T statistic for each local maximum. P<0.01 based on non-stationary cluster-extent False discovery rate correction.

Supplementary Table 10. Structural covariance network for brain-derived neurotrophic factor Met/Met homozygotes with right dorsolateral prefrontal cortex as seed.

Main Cluster	Peak regions	Stereotaxic coordinates				Extent	Max T	P-value
		Side	x	y	z			
**Middle Frontal**		**R**	**44**	**36**	**19**	**39656**	**16.84**	**<0.001**
	Frontal inferio-triangular region	R	45	26	30	s.c.	9.94	<0.001
	Frontal inferio-triangular region	L	-41	35	7	s.c.	7.7	<0.001
**Middle Temporal**		**R**	**53**	**-64**	**15**	**4015**	**5.66**	**<0.001**
	Inferior Parietal	R	57	-43	46	s.c.	4.81	<0.001
	Inferior Parietal	R	50	-49	48	s.c.	4.41	<0.001
**Inferior Temporal**		**L**	**-51**	**-21**	**-26**	**10964**	**5.61**	**<0.001**
	Fusiform	L	-39	-42	-20	s.c.	5.27	<0.001
	Middle Temporal	L	-60	-39	-8	s.c.	5.12	<0.001
**Supp_Motor_Area**		**L**	**-11**	**3**	**46**	**797**	**5.27**	**<0.001**
	Middle Cingulum	L	-11	26	31	s.c.	4.29	<0.001
**Thalamus**		**L**	**-18**	**-28**	**6**	**761**	**4.68**	**<0.001**
	Thalamus	L	-12	-19	9	s.c.	3.88	<0.001
	Thalamus	L	-5	-15	3	s.c.	3.24	0.001
**Calcarine**		**L**	**-11**	**-70**	**16**	**358**	**4.4**	**<0.001**
**Anteiror Cingulum**		**R**	**11**	**35**	**25**	**392**	**4.32**	**<0.001**
**Cuneus**		**R**	**6**	**-90**	**30**	**243**	**4.13**	**<0.001**
	Occipital_Sup	R	21	-91	27	s.c.	3.61	<0.001
	Cuneus	L	-3	-87	36	s.c.	3.16	0.001
**Middle Temporal**		**R**	**56**	**-9**	**-23**	**575**	**4.03**	**<0.001**
	Inferior Temporal	R	54	-11	-32	s.c.	3.97	<0.001
**Thalamus**		**R**	**9**	**-25**	**13**	**214**	**3.54**	**<0.001**
	Thalamus	R	14	-18	10	s.c.	3.46	0.001
	Thalamus	R	17	-28	0	s.c.	3.44	0.001
**Postcentral**		**R**	**38**	**-34**	**60**	**100**	**3.46**	**0.001**

Peak regions are within the Main cluster; R=right, L=left, s.c= same cluster
Max T is the maximum T statistic for each local maximum. P<0.01 based on non-stationary cluster-extent False discovery rate correction.

Supplementary Table 11. Structural covariance network for brain-derived neurotrophic factor Met/Val allele with right dorsolateral prefrontalcortex as seed.

Main Cluster	Peak regions	Stereotaxic coordinates				Extent	Max T	P-value
		Side	x	y	z			
**Middle Frontal**		**R**	**42**	**36**	**19**	**11657**	**20.82**	**<0.001**
	Frontal inferio triangular region	R	45	32	7	s.c.	6.35	<0.001
	Middle Frontal	R	44	29	37	s.c.	5.75	<0.001
**Middle orbital Frontal**		**L**	**-38**	**45**	**-8**	**21997**	**6.94**	**<0.001**
	Inferior orbital Frontal	L	-39	36	-11	s.c.	6.65	<0.001
	Frontal inferio triangular region	L	-41	38	3	s.c.	6.17	<0.001
**Inferior Parietal**		**R**	**33**	**-54**	**54**	**371**	**4.46**	**<0.001**
**SupraMarginal**		**L**	**-60**	**-27**	**18**	**591**	**4.34**	**<0.001**
**Cerebelum_8**		**R**	**8**	**-61**	**-32**	**285**	**4.22**	**<0.001**
**Inferior Temporal**		**L**	**-54**	**-37**	**-23**	**166**	**4.09**	**<0.001**
**Middle Cingulum**		**R**	**9**	**5**	**37**	**201**	**4.06**	**<0.001**
**Cerebelum_6**		**R**	**44**	**-47**	**-29**	**347**	**3.99**	**<0.001**
	Inferior Temporal	R	51	-51	-27	s.c.	3.78	<0.001
**Superior Frontal**		**R**	**23**	**24**	**52**	**105**	**3.92**	**<0.001**

Peak regions are within the Main cluster; R=right, L=left, s.c= same cluster
Max T is the maximum T statistic for each local maximum. P<0.01 based on non-stationary cluster-extent False discovery rate correction.

Supplementary Table 12. Structural covariance network brain-derived neurotrophic factor Val/Val homozygotes with right dorsolateral prefrontal cortex as seed.

Main Cluster	Peak regions	Stereotaxic coordinates				Extent	Max T	P-value
		Side	x	y	z			
**Middle Frontal**		**R**	**44**	**35**	**21**	**8137**	**17.4**	**<0.001**
	Middle Frontal	R	32	20	49	s.c.	6.05	<0.001
	Middle orbital Frontal	R	41	48	-14	s.c.	5.65	<0.001
**Middle Frontal**		**L**	**-24**	**53**	**18**	**7564**	**6.75**	**<0.001**
	Superior Frontal	L	-20	8	57	s.c.	6.73	<0.001
	Precentral	L	-45	2	24	s.c.	6.32	<0.001
**Anteiror Cingulum**		**R**	**9**	**24**	**27**	**1060**	**4.94**	**<0.001**
	Middle Cingulum	R	8	3	34	s.c.	4.32	<0.001
	Anteiror Cingulum	R	12	47	7	s.c.	3.93	<0.001
**Middle Temporal**		**R**	**48**	**-61**	**22**	**269**	**4.62**	**<0.001**
	Middle Temporal	R	54	-49	21	s.c.	3.6	<0.001
**Rolandic Operculum**		**R**	**51**	**-9**	**10**	**1162**	**4.52**	**<0.001**
	Insula	R	41	-16	10	s.c.	4.36	<0.001
**Superior medial Frontal**		**R**	**5**	**60**	**12**	**121**	**4.49**	**<0.001**
**Thalamus**		**R**	**11**	**-16**	**0**	**119**	**4.45**	**<0.001**
**Inferior Parietal**		**L**	**-36**	**-52**	**40**	**105**	**3.99**	**<0.001**
**Cuneus**		**L**	**-11**	**-78**	**28**	**161**	**3.98**	**<0.001**
	Calcarine	L	-11	-67	22	s.c.	3.65	<0.001
**Middle Temporal**		**L**	**-54**	**-18**	**-11**	**139**	**3.93**	**<0.001**
**Middle Temporal**		**L**	**-51**	**-48**	**18**	**126**	**3.85**	**<0.001**
	Middle Temporal	L	-51	-39	9	s.c.	3.72	<0.001

Peak regions are within the Main cluster; R=right, L=left, s.c= same cluster
Max T is the maximum T statistic for each local maximum. P<0.01 based on non-stationary cluster-extent False discovery rate correction.

Supplementary Table 13. Connectivity differences between brain-derived neurotrophic factor genotypes with left posterior cingulate as seed

Main Cluster	Peak regions	Stereotaxic coordinates				Extent	Max T	P-value
		Side	x	y	z			
Met/Val>Met/Met								
Inferior Temporal		R	39	-64	-9	168	3.35	0.001
Precentral		L	-36	2	42	195	3.17	0.001
Val/Val>Met/Met								
Anteiror Cingulum		L	-6	44	13	116	2.6	0.005
	Superior medial Frontal	L	-12	38	19	s.c.	2.47	0.008

Peak regions are within the Main cluster; R=right, L=left, s.c= same cluster
Max T is the maximum T statistic for each local maximum. P<0.01 based on non-stationary cluster-extent False discovery rate correction.

Supplementary Ttable 14. Structural covariance network for with right frontoinsularcortex as seed

Main Cluster	Peak regions	Side	Stereotaxic coordinates x	y	z	Extent	Max T	P-value
Met/Val>Met/Met								
Middle orbital Frontal		R	33	48	-2	183	3.19	0.001
	Inferior orbital Frontal	R	39	39	-3	s.c.	2.79	0.003
Insula		L	-26	23	9	126	2.94	0.002
Val/Val>Met/Met								
NA								

Peak regions are within the Main cluster; R=right, L=left, s.c= same cluster
Max T is the maximum T statistic for each local maximum. P<0.01 based on non-stationary cluster-extent False discovery rate correction.

Supplementary Table 15. Structural covariance network for with right dorsolateral prefrontal as seed

Main Cluster	Peak regions		Stereotaxic coordinates		Extent	Max T	P-value
		Side	x	y	z			
GA>AA								
NA								
GG>AA								
Cerebelum_6		L	-23	-60	-33	450	3.46	<0.001
	Cerebelum_Crus_1	L	-30	-60	-38	s.c.	3.06	0.001
Cerebelum_4_5		L	-18	-30	-29	772	3.42	<0.001
	Cerebelum_4_5	L	-24	-33	-33	s.c.	3.25	0.001
Hippocampus		R	41	-30	-9	259	3.3	0.001
Postcentral		R	44	-30	37	128	3.18	0.001
Caudate		L	-15	5	18	279	2.8	0.003

Peak regions are within the Main cluster; R=right, L=left, s.c= same cluster
Max T is the maximum T statistic for each local maximum. P<0.01 based on non-stationary cluster-extent False discovery rate correction.
